# Meaningful differences and changes for five Patient‐Reported Outcomes Measurement Information System domains in a large cohort of patients with cancer

**DOI:** 10.1002/cncr.70219

**Published:** 2025-12-18

**Authors:** Karen Llave, Jiwon Kim, Maja Kuharic, Kathryn Jackson, Nicola Lancki, Kimberly A. Webster, David Cella

**Affiliations:** ^1^ Department of Medical Social Sciences Northwestern University Feinberg School of Medicine Chicago Illinois USA; ^2^ Department of Preventive Medicine Northwestern University Feinberg School of Medicine Chicago Illinois USA

**Keywords:** cancer, patient‐reported outcomes, Patient‐Reported Outcomes Measurement Information System (PROMIS), quality of life

## Abstract

**Background:**

Patient‐reported outcomes are valuable sources of information in research and clinical care, particularly in populations experiencing long‐term effects on health‐related quality of life such as patients with cancer. Identifying meaningful differences and changes in patient‐reported outcomes can prove useful in research and clinical settings.

**Methods:**

Data from a system‐wide symptom‐management intervention trial were analyzed. Patients with cancer completed Patient‐Reported Outcomes Measurement Information System (PROMIS) computer‐adaptive tests in pain, fatigue, depression, anxiety, and physical function at baseline and 6 months. The authors used a combination of distribution‐based and anchor‐based approaches to estimate meaningful differences in PROMIS scores at baseline. Meaningful change estimates were calculated using mean changes in PROMIS scores in response to selected anchors.

**Results:**

The sample (*n* = 3907) was predominantly female (66.1%) and non‐Hispanic (84.2%). Overall, the sample reported PROMIS scores close to the general population standard (mean ± standard deviation score, 50 ± 10). Longitudinal anchor‐based estimates ranged from 2.4 to 8.0 points for pain interference, 2.0–4.4 points for depression, 2.2–4.5 points for anxiety, 2.0–6.3 points for fatigue, and 1.9–4.4 points for physical functioning. Longitudinal, anchor‐based findings revealed smaller changes than cross‐sectional differences indicating the responsiveness of PROMIS measures to patient‐reported meaningful changes over time. Supporting distribution‐based information using SEM criteria produced generally smaller values, ranging from 1.14 for depression to 2.14 for fatigue.

**Conclusions:**

PROMIS computer‐adaptive test measures of pain, fatigue, anxiety, depression, and physical function are sensitive to group differences and changes over time. Regular symptom monitoring in conjunction with the recommended thresholds could be useful in research and clinical settings.

## Abstract 2

PROMIS (Patient‐Reported Outcomes Measurement Information System) computer‐adaptive testing measures of pain, fatigue, anxiety, depression, and physical function are sensitive to group differences and changes over time. Regular symptom monitoring in conjunction with the recommended thresholds could be useful in research and clinical settings.

## INTRODUCTION

Patient‐reported outcomes (PROs) deliver valuable insight that can support decision making in both research and clinical care. The Patient‐Reported Outcomes Measurement Information System (PROMIS) offers item banks for measuring multiple health domains, such as pain, fatigue, anxiety, depression, and physical function. Items in these banks are calibrated to enable flexible, fixed‐form and computer‐adaptive testing (CAT).^1^, ^2^, ^3^ PROMIS domain scores are expressed as *T scores* and typically are referenced to a general population, with a mean ± standard deviation (SD) score of 50 ± 10. Assessing the meaningfulness of *group* differences or changes in these domains is crucial for interpreting research findings. Similarly, understanding the magnitude of *individual* change that is likely to be meaningful to patients is useful for guiding clinical decision making.

Most experts recommend the use of relevant and meaningful anchors to help estimate *meaningful differences* (MDs) and *meaningful changes* (MCs).^4^, ^5^, ^6^, ^7^ Previous research in this area with various PROMIS domains has produced estimates for MD and MC scores ranging from 3 to 10 points.^8^, ^9^, ^10^, ^11^, ^12^, ^13^, ^14^ These estimates are not fixed properties of instruments; they can vary depending on the context of the specific application, including diagnosis, prognosis, treatment options, and patient factors. The cancer treatment setting is an area of rapid growth of interest in PROMIS application.^15^, ^16^, ^17^ To that end, we conducted a large, health system–wide implementation of PROMIS CAT measures of pain, fatigue, anxiety, depression, and physical function.^18^, ^19^ We included several anchors that would allow us to further our understanding of MDs and MCs in these five PROMIS domains, specifically in the context of cancer care. The current study describes our anchor‐based methods to establish MDs and MCs in multiple PROMIS domains.

## MATERIALS AND METHODS

Patients were recruited through a system‐wide symptom‐management intervention implemented in 30 outpatient oncology clinics across seven regional clusters within the Northwestern Medicine health care system.^18^ Recruitment and data collection occurred between August 2020 and April 2024.^19^ Eligible patients (1) were at least 18 years old, (2) had a solid tumor or hematologic malignant neoplasm diagnosis in their medical record within the past 10 years, (3) had visited a recruiting clinic within the past year, (4) were able to read English or Spanish, and (5) had a valid email address. Study procedures were approved by the Northwestern University institutional Review Board (STU00208413), and informed consent was obtained from all participants. All analyses were conducted using R (version 4.4.0; R Core Team, 2024).

### Procedure

Participants were invited to complete measures through a Research Electronic Data Capture (REDCap) survey over a 12‐month period. At baseline, participants completed all PROMIS CATs and additional anchor measures. PROMIS CATs and measures from the PROs version of the Common Terminology Criteria for Adverse Events (PRO‐CTCAE)^20^ were completed monthly, whereas all other measures were completed quarterly. Sociodemographic and clinical variables were self‐reported or extracted from electronic medical records. Baseline and 6‐month data were analyzed for this report.

### Measures

Participants completed five PROMIS CATs: Pain Interference (version 1.1), Depression (version 1.0), Anxiety (version 1.0), Fatigue (version 1.0), and Physical Function (version 1.2). The CAT versions of the PROMIS measures use stopping rules to offer brief, tailored items for each respondent and more precise measurement of symptom/function severity. Each CAT used stopping rules that ended administration once the standard error fell below 0.3 or upon reaching the 12‐item maximum. Higher scores on a PROMIS measure indicate higher levels of the concept being measured. For example, higher PROMIS‐Anxiety scores indicate more anxiety, and higher PROMIS‐Physical Function scores indicate better physical function.

Participants completed selected items of the following additional measures quarterly: the Consumer Assessment of Healthcare Providers and Systems (CAHPS) Cancer Care Survey (whether the care team advised or helped the patient with pain, fatigue, emotional problems),^21^ the PROs version of the Common Terminology Criteria for Adverse Events (PRO‐CTCAE; severity, frequency, and/or interference of nausea, vomiting, diarrhea, constipation, shortness of breath, and insomnia),^20^ the Functional Assessment of Cancer Therapy‐General 7 (FACT‐G7; a brief, seven‐item measure reflecting the highest priority concerns across key domains of cancer‐related quality of life),^22^ and the University of California‐Los Angeles Loneliness Scale (single item on isolation).^23^

### Anchor‐based approach

We used anchor‐based methods to identify cross‐sectional MDs and longitudinal MCs. Cross‐sectional methods compare patients' PROMIS scores based on clinically distinct groups as defined by patients' responses to selected anchors. Longitudinal methods involve comparing changes in the PROMIS measures with changes in patient‐reported anchors over time. This analysis includes self‐reported data on 11 clinically relevant anchors reflecting physical (e.g., pain, fatigue), emotional (e.g., loneliness), and overall well‐being (e.g., enjoyment of life, social function) domains. Table S1 summarizes correlations between PROMIS outcome measures and the anchors used to estimate MDs (see Supporting Information). Anchors were selected based on clinical relevance. Based on prior work, we set 0.37 as the minimum required correlation between an anchor and a PROMIS measure.^27^

### Cross‐sectional anchor‐based analysis

In cross‐sectional analysis, patients were categorized into clinically distinct groups as defined by their responses to the selected anchors. For anchors that were measured ordinally, each response option was defined as a clinically distinct group. Groups were defined using cutoffs established in prior work.^28^ Mean differences between adjacent groups were calculated and represent estimates of the MD. Effect sizes were computed by dividing these mean differences by the overall SD for the sample.^29^

### Longitudinal anchor‐based analysis

For longitudinal analysis, anchor data were measured at baseline (T0) and at 6 months (T1). For ordinal anchors, we defined a one‐category change (either positive or negative) as meaningful.^30^ We defined an MC on the FACT‐G7 (scale range, 0–28) as at least a three‐point difference in either direction. Based on changes in scores from T0 to T1, patients were categorized into *better*, *worse*, or *about the same* groups. Mean differences in PROMIS scores between the *better/worse* groups and the *about the same* group were deemed estimates of the MD. Longitudinal effect sizes were calculated by dividing each group's mean PROMIS change score by the sample's overall SD at T1.

### Distribution‐based approach

Three distribution‐based methods were used to estimate MDs in PROMIS measures. Based on existing work suggesting one half of the as a likely approximation of a medium size effect and one fifth of the SD estimating a small effect size, we calculated one third and one half of the SD to supplement information gathered from anchor‐based results.^24^, ^25^ We also computed 1 standard error of measurement (SEM) for each of the five PROMIS measures at baseline and at 6 months. The SEM was computed as SEM = SD * √(1 − *r*), where SD is the standard deviation of the measure, and *r* is the reliability of the measure (computed as 1 − mean [standard error]^2^).^26^

## RESULTS

The study team reached out to 39,952 patients, resulting in 4104 consented participants (10.3% recruitment rate). Among those enrolled, 95.2% (*n* = 3907) completed one or more PROMIS measure(s) at baseline and were included in this analysis. Sample characteristics at baseline and at 6 months and baseline PROMIS scores for this sample are summarized in Table 1. At baseline, the average time since diagnosis was 3.7 months. The most common cancer types were breast cancer (*n* = 1254; 30.6%) and lymphoma (*n* = 476; 11.6%). Most participants were diagnosed with stage I (*n* = 1118; 40.4%) or stage II (*n* = 556; 20.1%) disease. Approximately 71.8% (*n* = 2806) of the baseline sample completed one or more PROMIS measure(s) at 6 months and were included in longitudinal data analyses. Participants' average age was 60.5 years, and most were women (66.1%) and non‐Hispanic (84.2%). Mean PROMIS scores at baseline and at 6 months are reported in Table 1. Participants reported decreases in depression, anxiety, and fatigue at 6 months, whereas pain interference and physical function levels remained relatively stable.

**TABLE 1:** Sample characteristics.

		No. (%)	
Characteristic		Baseline, *n* = 3907	6‐month follow‐up, *n* = 2806
Sex at birth			
Male		1326 (33.9)	958 (34.1)
Female		2581 (66.1)	1848 (65.9)
Race			
American Indian/Alaskan Native		9 (0.2)	6 (0.2)
Asian		99 (2.5)	71 (2.5)
Black/African American		200 (5.1)	115 (4.1)
Native Hawaiian/Pacific Islander		4 (0.1)	3 (0.1)
White		3291 (84.2)	2411 (85.9)
Unknown/not reported		304 (7.8)	200 (7.1)
Ethnicity			
Not Hispanic		3291 (84.2)	2403 (85.6)
Hispanic		158 (4.0)	102 (3.6)
Unknown or not reported		458 (11.7)	301 (10.7)
Education			
Less than high school		17 (0.4)	6 (0.2)
Some high school		15 (0.4)	8 (0.2)
High school or equivalent/GED		234 (6.0)	151 (5.6)
Some college: Technical/associates		886 (22.7)	601 (22.5)
College, Bachelors		1081 (27.7)	784 (29.3)
Some graduate school		223 (5.7)	169 (6.3)
Graduate school/advanced degree		1250 (32.0)	950 (35.5)
Prefer not to answer/missing		201 (5.1)	137 (4.9)
Insurance			
Medicare only		240 (6.4)	187 (7.0)
Medicare + supplemental insurance		1257 (33.8)	979 (36.6)
Medicaid		127 (3.4)	69 (2.6)
Private		1997 (53.6)	1377 (51.4)
Uninsured or self‐pay		26 (0.7)	18 (0.7)
Military or veterans sponsored		23 (0.6)	19 (0.7)
Don’t know/missing		237 (6.1)	157 (5.6)
Beacon treatment continuum			
Curative		766 (19.6)	564 (20.1)
Noncurative		454 (11.6)	318 (11.3)
Survivor		2687 (68.8)	1924 (68.6)

**PROMIS domain**	**No. at baseline/6 months)**	**Baseline mean ± SD**	**6‐month mean ± SD**
Pain interference	3907/2806	49.9 ± 9.7	49.1 ± 9.8
Depression	3874/2778	49.0 ± 8.4	47.1 ± 8.9
Anxiety	3859/2766	51.2 ± 9.3	48.9 ± 9.6
Fatigue	3891/2789	50.3 ± 10.5	49.6 ± 10.6
Physical function	3845/2754	46.8 ± 8.8	46.5 ± 8.7

Abbreviations: GED, general educational development; PROMIS, Patient‐Reported Outcomes Measurement Information System; SD, standard deviation.

### Cross‐sectional and longitudinal, anchor‐based meaningful differences

Anchors were selected based on a conceptual match and having a correlation of ≥0.37 between the selected anchor and the PROMIS domain,^7^, ^30^ as summarized in Table S2. Selected anchors items, response options, and corresponding ranges and distributions are detailed in Table S3. We identified from three to seven suitable anchors for each PROMIS domain. Cross‐sectional MD estimates and effect sizes are reported in the upper one half of Tables 2, 3, 4, 5, and 6. Average cross‐sectional MD estimates ranged from 3.5 points for anxiety to 6.0 points for pain interference and are summarized in detail in Table S4. These are plotted alongside distribution‐based and longitudinal, anchor‐based MD estimates in the Supporting Information (see Figure S5). Across all domains, cross‐sectional MD estimates were larger in magnitude and had moderate to larger effect sizes compared with longitudinal estimates. Longitudinal MDs were smaller and more variable, often with smaller effect sizes, particularly among the patient groups that reported doing better at 6 months.

**TABLE 2:** Patient Reported Outcomes Measurement Information System–Pain interference meaningful difference estimates by method and anchor variable.

Anchor variable	Comparison	MD estimate ± SE	Effect size
Cross‐sectional			
CAHPS: Pain	No vs. yes	9.3 ± 0.11	0.96
FACT‐G7: Pain	Not at all vs. a little bit	10.3 ± 0.09	1.1
	A little bit vs. somewhat	5.8 ± 0.13	0.60
	Somewhat vs. quite a bit	4.7 ± 0.17	0.48
	Quite a bit vs. very much	4.9 ± 0.27	0.50
FACT‐G7: Overall	>22 vs. 19–22	5.2 ± 0.12	0.54
	19–22 vs. 16–18	4.2 ± 0.14	0.43
	16–18 vs. 13–15	3.6 ± 0.18	0.37
	13–15 vs. <13	5.8 ± 0.20	0.60
	Mean [range]	6.0 [3.6–10.3]	0.62 [0.37–1.1]
Longitudinal (Ref: About the same)			
CAHPS: Pain	Worse	3.7 ± 0.19	0.34
	Better	−2.4 ± 0.20	−0.29
FACT‐G7: Pain	Worse	8.0 ± 0.17	0.79
	Better	−7.6 ± 0.18	−0.79
FACT‐G7: Overall	Worse	6.1 ± 0.25	0.64
	Better	−5.0 ± 0.22	−0.48
	Mean [range]	5.5 [2.4–8.0]	0.56 [0.29–0.79]

Abbreviations: CAHPS, Consumer Assessment of Healthcare Providers and Systems; FACT‐G7, Functional Assessment of Cancer Therapy‐General 7; MD, meaningful difference; Ref, reference category; SE, standard error; UCLA, University of California‐Los Angeles.

**TABLE 3:** Patient‐Reported Outcomes Measurement Information System–Depression meaningful difference estimates by method and anchor variable.

Anchor variable	Comparison	MD estimate ± SE	Effect size
Cross‐sectional			
CAHPS: Emotional problems	No vs. yes	7.8 ± 0.10	0.93
FACT‐G7: Able to enjoy life	Very much vs. quite a bit	6.1 ± 0.10	0.73
	Quite a bit vs. somewhat	5.1 ± 0.11	0.61
	Somewhat vs. a little bit	2.9 ± 0.21	0.34
	A little bit vs. not at all	−1.6 ± 0.52	−0.19
FACT‐G7: Content with life	Very much vs. quite a bit	5.1 ± 0.11	0.61
	Quite a bit vs. somewhat	4.6 ± 0.11	0.55
	Somewhat vs. a little bit	2.9 ± 0.17	0.34
	A little bit vs. not at all	1.3 ± 0.25	0.15
UCLA: Loneliness	Rarely vs. never	6.4 ± 0.11	0.76
	Sometimes vs. rarely	4.2 ± 0.11	0.50
	Usually vs. sometimes	3.8 ± 0.24	0.45
	Always vs. usually	0.8 ± 0.54	0.10
FACT‐G7: Overall	>22 vs. 19–22	6.0 ± 0.11	0.71
	19–22 vs. 16–18	3.3 ± 0.13	0.39
	16–18 vs. 13–15	2.4 ± 0.16	0.29
	13–15 vs. <13	3.2 ± 0.18	0.38
	Mean [range]	4.0 [0.80–7.8]	0.47 [0.10–0.93]
Longitudinal (Ref: About the same)			
CAHPS: Emotional problems	Worse	2.0 ± 0.20	0.08
	Better	−2.6 ± 0.17	−0.44
FACT‐G7: Able to enjoy life	Worse	4.1 ± 0.15	0.30
	Better	−3.5 ± 0.15	−0.55
FACT‐G7: Content with life	Worse	2.8 ± 0.15	0.18
	Better	−3.3 ± 0.14	−0.51
UCLA: Loneliness	Worse	3.1 ± 0.15	0.21
	Better	−3.4 ± 0.15	−0.52
FACT‐G7: Overall	Worse	4.1 ± 0.18	0.36
	Better	−4.4 ± 0.14	−0.59
	Mean [range], absolute value	3.3 [2.0–4.4]	0.37 [0.08–0.59]

Abbreviations: CAHPS, Consumer Assessment of Healthcare Providers and Systems; FACT‐G7, Functional Assessment of Cancer Therapy‐General 7; MD, meaningful difference; Ref, reference category; SE, standard error; UCLA, University of California‐Los Angeles.

**TABLE 4:** Patient‐Reported Outcomes Measurement Information System–Anxiety meaningful difference estimates by method and anchor variable.

Anchor variable	Comparison	MD Estimate ± SE	Effect size
Cross‐sectional			
CAHPS: Emotional problems	No vs. yes	9.2 ± 0.10	0.99
FACT‐G7: Worry condition will worsen	Not at all vs. a little bit A little bit vs. somewhat Somewhat vs. quite a bit Quite a bit vs. very much	4.3 ± 0.13 3.8 ± 0.13 4.1 ± 0.17 2.2 ± 0.21	0.46 0.41 0.44 0.24
FACT‐G7: Able to enjoy life	Very much → quite a bit	6.3 ± 0.11	0.68
	Quite a bit → somewhat	4.9 ± 0.12	0.53
	Somewhat → a little bit	2.3 ± 0.22	0.25
	A little bit → not at all	−1.0 ± 0.53	−0.11
FACT‐G7: Content with life	Very much → quite a bit	5.5 ± 0.12	0.59
	Quite a bit → somewhat	4.0 ± 0.12	0.43
	Somewhat → a little bit	3.0 ± 0.18	0.32
	A little bit → not at all	1.1 ± 0.26	0.12
UCLA: Loneliness	Rarely → never	5.9 ± 0.12	0.64
	Sometimes → rarely	4.4 ± 0.12	0.48
	Usually → sometimes	3.7 ± 0.25	0.40
	Always → usually	0.3 ± 0.52	0.03
FACT‐G7: Overall	>22 → 19–22	6.1 ± 0.12	0.66
	19–22 → 16–18	3.9 ± 0.14	0.42
	16–18 → 13–15	2.4 ± 0.17	0.26
	13–15 → <13	3.5 ± 0.20	0.38
	Mean [range]	3.9 [0.30–9.2]	0.42 [0.03–0.99]
Longitudinal (Ref: About the same)			
CAHPS: Emotional problems	Worse	2.2 ± 0.21	0.06
	Better	−2.9 ± 0.18	−0.47
FACT‐G7: Worry condition will worsen	Worse	2.5 ± 0.16	0.10
	Better	−2.4 ± 0.14	−0.41
FACT‐G7: Able to enjoy life	Worse	3.8 ± 0.16	0.21
	Better	−3.6 ± 0.15	−0.55
FACT‐G7: Content with life	Worse	2.6 ± 0.16	0.11
	Better	−3.3 ± 0.15	−0.50
UCLA: Loneliness	Worse	2.5 ± 0.16	0.09
	Better	−2.8 ± 0.15	−.046
FACT‐G7: Overall	Worse	4.5 ± 0.19	0.33
	Better	−4.5 ± 0.15	−0.60
	Mean [range]	3.1 [2.2–4.5]	0.32 [0.06–0.60]

Abbreviations: CAHPS, Consumer Assessment of Healthcare Providers and Systems; FACT‐G7, Functional Assessment of Cancer Therapy‐General 7; MD, meaningful difference; Ref, reference category; SE, standard error; UCLA, University of California‐Los Angeles.

**TABLE 5:** Patient‐Reported Outcomes Measurement Information System–Fatigue meaningful difference estimates by method and anchor variable.

Anchor variable	Comparison	MD estimate ± SE	Effect size
Cross‐sectional			
CAHPS: Change in energy	No vs. yes	10.0 ± 0.10	0.95
PRO‐CTCAE: Any severe	No vs. yes	10.8 ± 0.14	1.0
FACT‐G7: Able to enjoy life	Very much vs. quite a bit	7.00 ± 0.12	0.67
	Quite a bit vs. somewhat	6.1 ± 0.13	0.58
	Somewhat vs. a little bit	3.5 ± 0.23	0.33
	A little bit vs. not at all	−2.5 ± 0.55	−0.24
FACT‐G7: Lack of energy	Not at all vs. a little bit	6.4 ± 0.12	0.61
	A little bit vs. somewhat	5.5 ± 0.15	0.52
	Somewhat vs. quite a bit	3.2 ± 0.20	0.30
	Quite a bit vs. very much	4.4 ± 0.42	0.42
FACT‐G7: Content with life	Very much vs. quite a bit	5.9 ± 0.13	0.56
	Quite a bit vs. somewhat	5.8 ± 0.13	0.55
	Somewhat vs. a little bit	3.3 ± 0.19	0.31
	A little bit vs. not at all	3.3 ± 0.27	0.31
UCLA: Loneliness	Rarely vs. never	4.9 ± 0.13	0.47
	Sometimes vs. rarely	4.7 ± 0.14	0.45
	Usually vs. sometimes	3.8 ± 0.27	0.36
	Always vs. usually	2.8 ± 0.53	0.27
FACT‐G7: Overall	>22 vs. 19–22	7.8 ± 0.12	0.74
	19–22 vs. 16–18	4.9 ± 0.13	0.47
	16–18 vs. 13–15	4.4 ± 0.17	0.42
	13–15 vs. <13	4.6 ± 0.19	0.44
	Mean [range]	5.3 [2.5–10.8]	0.50 [0.24–1.0]
Longitudinal (Ref: About the same)			
CAHPS: Change in energy	Worse	4.0 ± 0.17	0.34
	Better	−3.2 ± 0.20	−0.33
PRO‐CTCAE: Any severe symptoms	Worse	5.1 ± 0.26	0.46
	Better	−2.5 ± 0.24	−0.26
FACT‐G7: Able to enjoy life	Worse	4.1 ± 0.17	0.38
	Better	−3.4 ± 0.16	−0.33
FACT‐G7: Lack of energy	Worse	6.0 ± 0.16	0.55
	Better	−5.8 ± 0.16	−0.56
FACT‐G7: Content with life	Worse	3.6 ± 0.16	0.32
	Better	−2.9 ± 0.15	−0.29
UCLA: Loneliness	Worse	3.1 ± 0.17	0.27
	Better	−2.0 ± 0.16	−0.21
FACT‐G7: Overall	Worse	6.3 ± 0.20	0.63
	Better	−5.3 ± 0.15	−0.46
	Mean [range]	4.1 [2.0–6.3]	0.39 [0.21–0.63]

Abbreviations: CAHPS, Consumer Assessment of Healthcare Providers and Systems; FACT‐G7, Functional Assessment of Cancer Therapy‐General 7; MD, meaningful difference; PRO‐CTCAE, Patient‐Reported Outcomes version of the Common Terminology Criteria for Adverse Events; Ref, reference category; SE, standard error; UCLA, University of California‐Los Angeles.

**TABLE 6:** Patient‐Reported Outcomes Measurement Information System–Physical function meaningful difference estimates by method and anchor variable.

Anchor variable	Comparison	MD estimate ± SE	Effect Size
Cross‐sectional			
FACT‐G7: Able to enjoy life	Very much vs. quite a bit	4.7 ± 0.11	0.53
	Quite a bit vs. Somewhat	4.6 ± 0.12	0.52
	Somewhat vs. a little bit	3.6 ± 0.22	0.41
	A little bit vs. not at all	−0.9 (.50)	−0.10
FACT‐G7: Content with life	Very much vs. quite a bit	3.6 ± 0.12	0.41
	Quite a bit vs. somewhat	4.5 ± 0.12	0.51
	Somewhat vs. a little bit	2.9 ± 0.17	0.33
	A little bit vs. not at all	2.7 ± 0.26	0.31
FACT‐G7: Lack of energy	Not at all vs. a little bit	4.6 ± 0.12	0.52
	A little bit vs. somewhat	4.6 ± 0.12	0.52
	Somewhat vs. quite a bit	3.3 ± 0.14	0.38
	Quite a bit vs. very much	5.0 ± 0.20	0.57
FACT‐G7: Pain	Not at all vs. a little bit	4.6 ± 0.11	0.52
	A little bit vs. somewhat	5.0 ± 0.14	0.57
	Somewhat vs. quite a bit	2.9 ± 0.19	0.33
	Quite a bit vs. very much	3.8 ± 0.31	0.43
FACT‐G7: Overall	>22 vs. 19–22	5.0 ± 0.12	0.57
	19–22 vs. 16–18	3.1 ± 0.14	0.35
	16–18 vs. 13–15	3.3 ± 0.17	0.38
	13–15 vs. <13	3.4 ± 0.19	0.39
	Mean [range]	3.8 [0.90–5.0]	0.43 [0.10–0.57]
Longitudinal (Ref: About the same)			
FACT‐G7: Able to enjoy life	Worse	−2.5 ± 0.16	−0.37
	Better	2.9 ± 0.15	0.25
FACT‐G7: Content with life	Worse	−1.9 ± 0.15	−0.30
	Better	2.3 ± 0.14	0.18
FACT‐G7: Lack of energy	Worse	−2.0 ± 0.14	−0.31
	Better	2.6 ± 0.15	0.22
FACT‐G7: Pain	Worse	−2.9 ± 0.15	−0.39
	Better	2.4 ± 0.15	0.21
FACT‐G7: Overall	Worse	−4.4 ± 0.18	−0.51
	Better	2.2 ± 0.14	0.24
	Mean [range]	2.6 [1.9–4.4]	0.30 [0.18–0.51]

Abbreviations: FACT‐G7, Functional Assessment of Cancer Therapy‐General 7; MD, meaningful difference; Ref, reference category; SE, standard error.

#### Distribution‐based information

A summary of distribution‐based MDs for the five PROMIS domains is provided in Table S2 (see Supporting Information). The reliability of each measure at baseline and at 6 months is reported in the first column. We estimated that small‐to‐medium effect sizes would correspond to score differences between 1.7 and 4.9 for pain interference, between 1.3 and 4.3 for depression, between 1.6 and 4.7 for anxiety, between 2.0 and 5.3 for fatigue, and between 1.4 and 4.4 for physical function. All mean standard errors were less than one third of the SD, suggesting good precision of the *T*‐score estimate.

### Domain‐specific patterns

#### Pain interference

Cross‐sectional MDs for pain interference ranged from 3.6 to 10.3. Longitudinal MDs were in the expected direction with patient groups who reported doing worse at 6 month, demonstrating positive MDs. Conversely, patient groups who reported doing better consistently had negative MDs.

#### Depression

Cross‐sectional MDs for depression were positive and moderately large, ranging from 0.80 to 7.8. One exception was observed among patient groups who reported being able to enjoy life *a little bit* versus *not at all*” at baseline (MD = −1.6). Longitudinal MDs were small (±4.0 points) and had lower effect sizes. Longitudinal MDs for patient groups who reported doing better at 6 months consistently had negative MDs.

#### Anxiety

Cross‐sectional MD estimates ranged from 0.30 to 9.2. We observed a similar exception among the high‐functioning patient group who reported being able to enjoy life *a little bit* versus *not at all* at baseline (MD = −1.0). Longitudinal change estimates were modest (±2.0–4.0 points) with lower effect sizes compared with cross‐sectional estimates.

#### Fatigue

We observed the highest cross‐sectional MD estimates within the fatigue domain—notably 10.0 and 10.8 with CAHPS and PRO‐CTCAE anchors, respectively. Similar to our findings in other domains, we observed a negative MD estimate among the high‐functioning patient group who reported being able to enjoy life *a little bit* versus *not at all* at baseline (MD = −2.5). Although smaller and with lower effect sizes, longitudinal estimates were in the expected direction, with negative MD estimates observed among the improved group.

#### Physical function

Cross‐sectional MD estimates for physical function were generally moderate and in the expected direction again with the exception of the group that reported being able to enjoy life *a little bit* versus *not at all* at baseline (MD = −0.9). Longitudinal MD estimates were positive for the improved group and negative for the worsening group, as expected. Longitudinal effect sizes were small, similar to what we observed across the other PROMIS domains.

## DISCUSSION

For the current study, we used distribution‐based and anchor‐based methods for estimating MDs and MCs in PROMIS scores among a sample of 3907 patients with cancer. Anchor‐based estimates were drawn from multiple anchors and ranged from 0.30 to 10.8 across all five domains. Although we focus on MDs and MCs, rather than minimally important differences and changes, it can reasonably be assumed that the minimally important values reside toward the low end of the reported estimates, with actual values determined by clinical context.^31^

Our distribution‐based (one third of the SD) estimates ranged from 2.77 for depression to 3.57 for fatigue. Across all domains, cross‐sectional MD estimates were larger in magnitude with higher effect sizes compared with longitudinal MD estimates. All MD estimates were in the direction expected with the one exception of those who reported being able to enjoy life a little bit versus not at all on the FACT‐G7.

Our findings support the utility of PROMIS measures in cancer care and provide thresholds for interpreting score differences in clinically distinct groups. Furthermore, these thresholds may also inform power calculations for future studies using PROMIS measures to examine patient‐reported quality of life. Our distribution‐based MD estimates using SEM criteria were notably small, ranging from 1.14 for depression to 2.15 for fatigue. However, this is likely a product of high Item Response Theory‐based reliabilities associated with the PROMIS CAT measures used in our study. Although we were able to detect small differences in groups, these are likely smaller than what is clinically meaningful. This highlights the distinction between statistical detectability and clinical relevance. The current study demonstrates that PROMIS CAT measures are sensitive enough capture subtle changes in symptom burden and physical function. However, the magnitude of these changes may not translate to meaningful effect in patients' daily lives. Therefore, these findings underscore both the analytic precision of PROMIS and the importance of anchoring quantitative change to patient‐experienced meaning.

In line with prior research, MCs varied by PROMIS domain. We observed larger and consistent change thresholds in fatigue and pain interference, whereas changes in psychosocial domains like depression and anxiety were smaller and more variable. This pattern suggests that psychosocial symptoms may fluctuate within a narrower range or may require more sustained shifts to be detected in patient scores.

Few studies have evaluated MDs in PROMIS measures with a robust sample of patients who have cander.^32^, ^33^, ^34^ Furthermore, we established responsiveness of the PROMIS measures with use of multiple clinically relevant anchors. However, these findings should be viewed considering some limitations. Although robust in size, the study sample was recruited from a single health system, primarily female and non‐Hispanic, highly educated, and privately insured, limiting potential generalizability of these findings to a more diverse setting or population. On average, our sample also reported PROMIS scores closer to general population standards (mean ± SD, 50 ± 10) than previously estimated reference values for patients with newly diagnosed cancer.^32^ Thus the recommended thresholds may not be suitable among more symptomatic patients.

Future studies should consider validation of PROMIS CATs in a diverse sample that would allow for exploration of thresholds by cancer type, treatment stage, or demographics. In addition, qualitative exploration of how patients interpret differences in their scores may be useful for patient–provider discussions related to symptom monitoring and shared decision making.

PROMIS measures demonstrate interpretable, clinically relevant MDs and MCs within the oncology setting. Routine assessment with PROMIS CATs, guided by evidence‐based thresholds, provides actionable information that can inform patient‐centered decision making in cancer care.

## AUTHOR CONTRIBUTIONS

**Karen Llave:** Conceptualization; writing—original draft; visualization; formal analysis; and writing—review and editing. **Jiwon Kim:** Writing—review and editing; and visualization. **Maja Kuharic:** Data curation; writing—review and editing; project administration; and visualization. **Kathryn Jackson:** Formal analysis; writing—review and editing; and visualization. **Nicola Lancki:** Data curation; writing—review and editing; and visualization. **Kimberly A. Webster:** Data curation; writing—review and editing; project administration; and visualization. **David Cella:** Conceptualization; funding acquisition; writing—review and editing; writing—original draft; methodology; data curation; supervision; and visualization.

## CONFLICT OF INTEREST STATEMENT

Maja Kuharic reports grants/contracts from the EuroQol Research Foundation and personal/consulting fees from AbbVie Inc. outside the submitted work. Kimberly Webster has ownership interest in FACIT.Org and FACIT.Trans outside the submitted work. The remaining authors disclosed no conflicts of interest.

## Supporting information

Supplementary Material

Supplementary Material

Supplementary Material

Supplementary Material

Supplementary Material

## Data Availability

The data that support the findings of this study will be openly available in the HealthMeasures Dataverse at https://dataverse.harvard.edu/dataverse/HealthMeasures upon publication.
