# Supplementary material for: Meaningful differences and changes for five Patient‐Reported Outcomes Measurement Information System domains in a large cohort of patients with cancer
Source: Cancer. 2025 Dec 18;132(1):e70219. doi: 10.1002/cncr.70219 (PMC12714130; doi:10.1002/cncr.70219)
Supplement: Supplementary file 1 — Supplementary Material [file CNCR-132-e70219-s005.docx]

| **Table S1.**  *Correlations between PROMIS domains and selected anchors at baseline* | | | | | |
| --- | --- | --- | --- | --- | --- |
|  | **PROMIS Domain** | | | | |
| **Anchor** | **Pain Interference** | **Depression** | **Anxiety** | **Fatigue** | **Physical Function** |
| *CAHPS* | | | | | |
| Pain | **-0.43** | -0.20 | -0.20 | -0.32 | 0.29 |
| Change in Energy | -0.32 | -0.24 | -0.23 | **-0.46** | 0.35 |
| Emotional Problems | -0.26 | **-0.44** | **-0.47** | -0.33 | 0.22 |
| *FACT-G7* |  |  |  |  |  |
| Overall | **-0.65** | **-0.63** | **-0.61** | **-0.73** | **0.59** |
| Able to Enjoy Life | -0.46 | **-0.55** | **-0.49** | **-0.52** | **0.47** |
| Content with Life | -0.48 | **-0.54** | **-0.48** | **-0.54** | **0.48** |
| Lack of Energy | -0.51 | -0.49 | -0.45 | **-0.81** | **0.59** |
| Pain | **-0.80** | -0.37 | -0.36 | -0.54 | **0.53** |
| Worry Condition Will Get Worse | -0.31 | -0.47 | **-0.49** | **-0.39** | 0.23 |
| PRO-CTCAE®  Any Severe Symptoms | 0.31 | 0.28 | 0.30 | **0.37** | -0.30 |
| UCLA Loneliness | 0.31 | **0.58** | **0.51** | **0.43** | -0.32 |
| CAHPS: Consumer Assessment of Healthcare Providers and Systems. FACT-G7: Functional Assessment of Cancer Therapy – General – 7-item version. PRO-CTCAE: Patient-Reported Outcomes version of the Common Terminology Criteria for Adverse Events  Note. *Bolded correlations indicate suitable anchors based on conceptual match and ≥ 0.37 correlation with PROMIS measure* | | | | | |
