# Supplementary material for: Meaningful differences and changes for five Patient‐Reported Outcomes Measurement Information System domains in a large cohort of patients with cancer
Source: Cancer. 2025 Dec 18;132(1):e70219. doi: 10.1002/cncr.70219 (PMC12714130; doi:10.1002/cncr.70219)
Supplement: Supplementary file 2 — Supplementary Material [file CNCR-132-e70219-s001.docx]

| **Table S2.**  *Summary of Distribution-based MD Estimates* | | | | |
| --- | --- | --- | --- | --- |
|  |  | **Criterion** | | |
|  | ***r*** | **⅓ SD** | **½ SD** | **SEM** |
| **Pain Interference** | | | | |
| Baseline  6-Months  Baseline to 6M | 0.98  0.97  - | 3.20  3.25  3.23 | 4.85  4.92  4.89 | 1.51  1.83  - |
| **Depression** | | | | |
| Baseline  6-Months  Baseline to 6M | 0.98  0.97  - | 2.77  2.92  2.88 | 4.20  4.43  4.37 | 1.14  1.49  - |
| **Anxiety** | | | | |
| Baseline  6-Months  Baseline to 6M | 0.98  0.97  - | 3.05  3.17  3.16 | 4.62  4.80  4.78 | 1.38  1.76  - |
| **Fatigue** | | | | |
| Baseline  6-Months  Baseline to 6M | 0.97  0.96  - | 3.47  3.51  3.57 | 5.26  5.31  5.41 | 1.77  2.14  - |
| **Physical Function** | | | | |
| Baseline  6-Months  Baseline to 6M | 0.98  0.97  - | 2.90  2.88  2.86 | 4.40  4.37  4.33 | 1.25  1.46  - |

*Note. r =* 1-mean(SE)^2^; SEM = σ_x_ √(1− *r*_x_)
