# Supplementary material for: Meaningful differences and changes for five Patient‐Reported Outcomes Measurement Information System domains in a large cohort of patients with cancer
Source: Cancer. 2025 Dec 18;132(1):e70219. doi: 10.1002/cncr.70219 (PMC12714130; doi:10.1002/cncr.70219)
Supplement: Supplementary file 3 — Supplementary Material [file CNCR-132-e70219-s003.docx]

| **Table S3.**  *Distribution and Ranges of Selected Anchors* | | | | | | | | | | |  |
| --- | --- | --- | --- | --- | --- | --- | --- | --- | --- | --- | --- |
| **Anchor** | **Min** | **1^st^ Qu.** | | | **Median** | | **Mean** | | **3^rd^ Qu.** | | **Max** |
| *CAHPS* | | | | | | | | | | |  |
| Pain | 1.0 | | 1.0 | 2.0 | | 1.7 | | 2.0 | | 2.0 | |
| Change in Energy | 1.0 | | 1.0 | 2.0 | | 1.6 | | 2.0 | | 2.0 | |
| Emotional Problems | 1.0 | | 2.0 | 1.7 | | 2.0 | | 2.0 | | 2.0 | |
| *FACT-G7* |  |  | | |  | |  | |  | |  |
| Overall | 0.0 | | 16.0 | 20.0 | | 19.5 | | 24.0 | | 28.0 | |
| Able to Enjoy Life | 0.0 | | 2.0 | 3.0 | | 2.9 | | 4.0 | | 4.0 | |
| Content with Life | 0.0 | | 2.0 | 3.0 | | 2.7 | | 4.0 | | 4.0 | |
| Lack of Energy | 0.0 | | 2.0 | 3.0 | | 2.4 | | 3.0 | | 4.0 | |
| Pain | 0.0 | | 2.0 | 3.0 | | 3.0 | | 4.0 | | 4.0 | |
| Worry Condition Will Get Worse | 0.0 | | 2.0 | 3.0 | | 2.5 | | 3.0 | | 4.0 | |
| PRO-CTCAE®  Any Severe Symptoms | 0.0 | | 0.0 | 0.0 | | 0.1 | | 0.0 | | 1.0 | |
| UCLA Loneliness | 1.0 | | 1.0 | 2.0 | | 2.0 | | 3.0 | | 5.0 | |
| CAHPS: Consumer Assessment of Healthcare Providers and Systems. FACT-G7: Functional Assessment of Cancer Therapy – General – 7-item version. PRO-CTCAE: Patient-Reported Outcomes version of the Common Terminology Criteria for Adverse Events | | | | | | | | | | |  |

| **Variable** | **Label** | **Options** |
| --- | --- | --- |
| pro_ctcae9a_ | In the last 7 days, how OFTEN did you have NAUSEA? | 1=Never  2=Rarely  3=Occasionally 4=Frequently  5=Almost Constantly |
| pro_ctcae9b_ | In the last 7 days, what was the SEVERITY of your NAUSEA at its WORST? | 1=None  2=Mild  3=Moderate  4=Severe  5=Very Severe |
| pro_ctcae10a_ | In the last 7 days, how OFTEN did you have VOMITING? | 1=Never  2=Rarely  3=Occasionally 4=Frequently  5=Almost Constantly |
| pro_ctcae10b_ | In the last 7 days, what was the SEVERITY of your VOMITING at its WORST? | 1=None  2=Mild  3=Moderate  4=Severe  5=Very Severe |
| pro_ctcae15a_ | In the last 7 days, what was the SEVERITY of your CONSTIPATION at its WORST? | 1=None  2=Mild  3=Moderate  4=Severe  5=Very Severe |
| pro_ctcae16_ | In the last 7 days, how OFTEN did you have LOOSE OR WATERY STOOLS (DIARRHEA/DIARRHOEA)? | 1=Never  2=Rarely  3=Occasionally 4=Frequently  5=Almost Constantly |
| pro_ctcae19a_ | In the last 7 days, what was the SEVERITY of your SHORTNESS OF BREATH at its WORST? | 1=None  2=Mild  3=Moderate  4=Severe  5=Very Severe |
| pro_ctcae19b_ | In the last 7 days, how much did your SHORTNESS OF BREATH INTERFERE with your usual or daily activities? | 1=None  2=A little bit 3=Somewhat  4=Quite a bit  5=Very Much |
| pro_ctcae52a_ | In the last 7 days, what was the SEVERITY of your INSOMNIA (including difficulty falling asleep, staying asleep, or waking up early) at its WORST? | 1=None  2=Mild  3=Moderate  4=Severe  5=Very Severe |
| pro_ctcae52b_ | In the last 7 days, how much did INSOMNIA (including difficulty falling asleep, staying asleep, or waking up early) INTERFERE with your usual or daily activities? | 1=None  2=A little bit 3=Somewhat  4=Quite a bit 5=Very Much |

**UCLA Loneliness**

Item: I feel Isolated from others

Response options: 1=Never 2=Rarely 3=Sometimes 4=Usually 5=Always

**CAHPS Items + Scoring**

Full questionnaire: <https://www.ahrq.gov/sites/default/files/wysiwyg/cahps/surveys-guidance/cancer/drug-eng-cancer-552a.pdf>

Scoring/domains measured: <https://www.ahrq.gov/sites/default/files/wysiwyg/cahps/surveys-guidance/cancer/measures-cancer-509.pdf>
