# Supplementary material for: Meaningful differences and changes for five Patient‐Reported Outcomes Measurement Information System domains in a large cohort of patients with cancer
Source: Cancer. 2025 Dec 18;132(1):e70219. doi: 10.1002/cncr.70219 (PMC12714130; doi:10.1002/cncr.70219)
Supplement: Supplementary file 4 — Supplementary Material [file CNCR-132-e70219-s004.docx]

| **Table S4.**  *Summary of cross-sectional and longitudinal anchor-based MD Estimates* | | | | |
| --- | --- | --- | --- | --- |
|  | **Cross-sectional** | | **Longitudinal** | |
|  | **Mean** | **Range** | **Mean** | **Range** |
| **Pain Interference**  **Depression**  **Anxiety**  **Fatigue**  **Physical Function** | **6.0**  **4.0**  **3.9**  **5.3**  **3.8** | **3.6 – 10.3**  **.80 – 7.8**  **.30 – 9.2**  **2.5 – 10.8**  **.90 – 5.0** | **5.5**  **3.3**  **3.1**  **4.1**  **2.6** | **2.4 – 8.0**  **2.0 – 4.4**  **2.2 – 4.5**  **2.0 – 6.3**  **1.9 – 4.4** |
