# Supplementary material for: Meaningful differences and changes for five Patient‐Reported Outcomes Measurement Information System domains in a large cohort of patients with cancer
Source: Cancer. 2025 Dec 18;132(1):e70219. doi: 10.1002/cncr.70219 (PMC12714130; doi:10.1002/cncr.70219)
Supplement: Supplementary file 5 — Supplementary Material [file CNCR-132-e70219-s002.docx]

**Figure S1.**
*Summary of MD Estimates*
